# Supplementary material for: Exploring New Ways to Analyze Data on the Spontaneous Physical Activity of Rodents Through a Weighing Balance
Source: Sensors (Basel). 2025 May 23;25(11):3290. doi: 10.3390/s25113290 (PMC12157805; doi:10.3390/s25113290)
Supplement: Supplementary file 1 [file sensors-25-03290-s001.zip › sensors-3597060-supplementary.pdf]

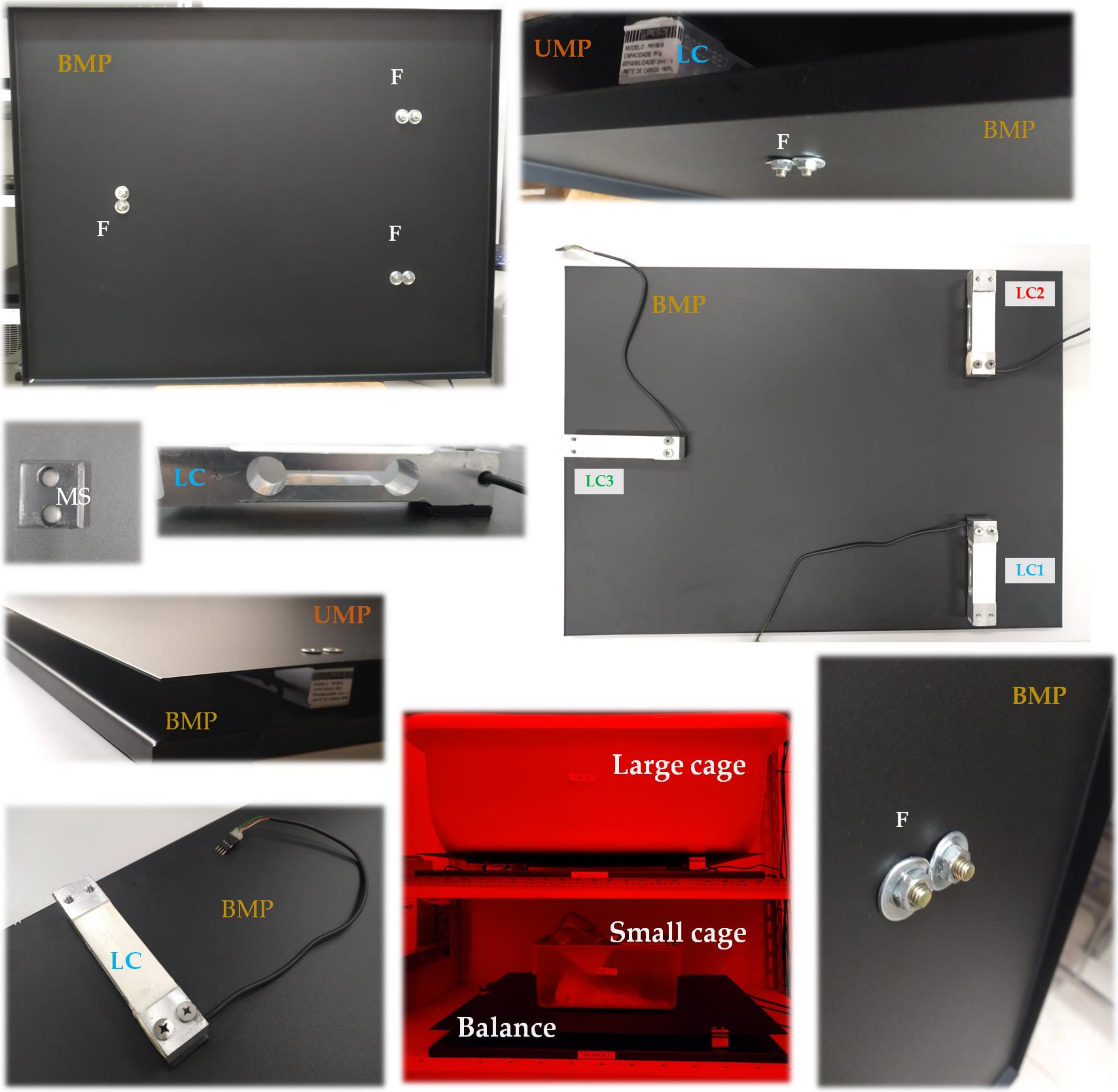

Figure S1: **Real photos of the weighing balance from different viewpoints**. Legend of Abbreviations: LC = Load cell, MS = Metal support, UMP = Upper metal plate on which cages are placed, BMP = Bottom metal plate in tray shape, F = Fasteners to ensure LC. There is also a picture showing the red lamps turned on only during animal handling.
